# Thermal shakedown in granular materials with irregular particle shapes

**DOI:** 10.1038/s41598-024-57503-2

**Published:** 2024-03-21

**Authors:** Yize Pan, Xiaohui Gong, Alessandro F. Rotta Loria

**Affiliations:** Department of Civil and Environmental Engineering, Subsurface Opportunities and Innovations Laboratory, Northwestern University, Evanston, USA

**Keywords:** Civil engineering, Soft materials, Condensed-matter physics, Geophysics

## Abstract

Granular materials with irregular particle shapes undergo a myriad of temperature variations in natural and engineered systems. However, the impacts of cyclic temperature variations on the mechanics of granular materials remain poorly understood. Specifically, little is known about the response of granular materials to cyclic temperature variations as a function of the following central variables: particle shape, applied stress level, relative density, and temperature amplitude. This paper presents advanced laboratory experiments to explore the impacts of cyclic temperature variations on the mechanics of granular materials, with a focus on sands. The results show that cyclic temperature variations applied to sands induce thermal shakedown: the accumulation of irreversible bulk deformations due to microstructural rearrangements caused by thermal expansions and contractions of the constituting particles. The deformation of sands caused by thermal shakedown strongly depends on particle shape, stress level, relative density, and temperature amplitude. This deformation is limited for individual thermal cycles but accumulates and becomes significant for multiple thermal cycles, leading to substantial compaction in sands and other granular materials, which can affect various natural and engineered systems.

## Introduction

Granular materials, including beads, granular food, and soils, continuously undergo temperature variations during their collection, processing, manufacturing, or handling. Given this evidence and the fact that temperature variations produce deformations in general substances, multiple studies have focused over the past two decades on the impacts of temperature variations on the mechanics of granular materials.

To date, the majority of investigations have studied granular materials with spherical particles^1–9^. These investigations have revealed that cyclic temperature variations yield thermal shakedown: the cumulative growth of plastic (i.e., irreversible) bulk deformations under constant applied stress due to thermal cycling, which shall stabilize as the number of loading–unloading cycles approaches infinity. Thermally induced particle deformations upon heating and cooling represent the origin of thermal shakedown. Experimental evidence shows that the bulk deformations of granular materials caused by thermal shakedown can be so significant that cyclic temperature variations may be used to densify such materials for storage purposes^1,4^. In fact, thermal shakedown involves effects in granular materials that are similar to those caused by vibration^10–12^ (despite being different in origin and nature). Numerical evidence further indicates that thermal shakedown could be harnessed to purposely tailor the properties of granular materials through structural changes^9^.

Besides granular materials with spherical and pseudo-spherical particles (e.g., beads), granular soils (e.g., sands) represent another ubiquitous class of particulate assemblies, which are omnipresent on Earth, the Moon, and Mars. Like other granular materials, sands undergo cyclic temperature variations in natural and engineered systems, typically in the range 10 °C ≤ $\Delta T$ ≤ 150 °C. From hundreds to thousands of thermal cycles are applied to sands during the lifetime of engineered systems^13–15^. Up to millions of thermal cycles are applied to sands in nature on Earth^16^, the Moon^17^, and Mars^18,19^. However, a key distinction that sets sands apart from granular materials with spherical particles is their inherently irregular particle *shape*. Particle shape provides sands and other granular materials with a highly complex fabric (i.e., the geometric arrangement of grains and voids) and a bulk behavior (e.g., thermo-hydro-mechanical), which crucially depend on the way particles with a given shape interact with each other^20^.

In recent years, thermally induced deformations of sands have been reported to play a central role in the failure of silos^21,22^, to represent a threat for the performance of thermal energy storage tanks^1,2,6–8,6,24^, to induce unwanted settlement of geothermal technologies^25–29^, and to trigger continuous changes in the morphology of landforms^30^. However, despite the ubiquity of sands and the foregoing observations, the mechanics of such materials subjected to thermal cycling remains poorly characterized and understood. Specifically, although one investigation has addressed some impacts of thermal cycles on sands^31^, all other studies available to date have focused on one thermal cycle at most^32–37^. In this context, several limitations characterize the state-of-the-art: firstly, there is a lack of studies investigating the role of particle shape in the development of thermal shakedown in sands and general granular materials. Additionally, there is a dearth of systematic studies on the influence of relative density and stress level on the development of thermal shakedown, despite these variables being known to govern the mechanics of granular materials under isothermal conditions. Lastly, there are no available studies on the effect of temperature amplitude on the mechanics of granular materials with irregular particle shapes. The current limited understanding of the mechanics of granular materials with irregular particle shapes subjected to thermal cycling implies an incapacity to thoroughly analyze and predict their response under non-isothermal conditions, which governs the performance of many engineered and natural systems.

This paper presents the results of advanced laboratory experiments with temperature control to unveil the impacts of multiple thermal cycles on the mechanics of granular materials as a function of particle shape, relative density, stress level, and temperature amplitude. Such an endeavor will focus on sands due to their inherently complex fabric. This work resorts to experiments performed under laterally restrained conditions to simulate the mechanics of sands at sites, aiming to replicate in-situ boundary conditions and minimize inconsistencies due to boundary effects, as discussed in previous studies for spherical beads^1,4^. The adopted sign convention in this work is the typical one of geomechanics and considers compressive stresses, contractive strains, and settlements as positive.

## Results

### Relative density and particle shape effects

This section starts analyzing the impacts of thermal cycles on the mechanics of granular materials with reference to the influence of relative density and particle shape. As in the remainder of this work, such an endeavor resorts to two types of tests: (1) isothermal tests involving mechanical loading up to a vertical stress of ${\sigma }_{v}=1$ MPa, followed by unloading at constant ambient temperature; and (2) non-isothermal tests involving an equivalent path of mechanical loading and unloading, with the additional application of 50 thermal cycles with a temperature amplitude of $\Delta T=60$ °C under a constant stress of ${\sigma }_{v}=60$ kPa. Loose and dense sands with rounded, subangular, and angular particle shapes are considered. See “Methods” for details.

**Figure 1:**
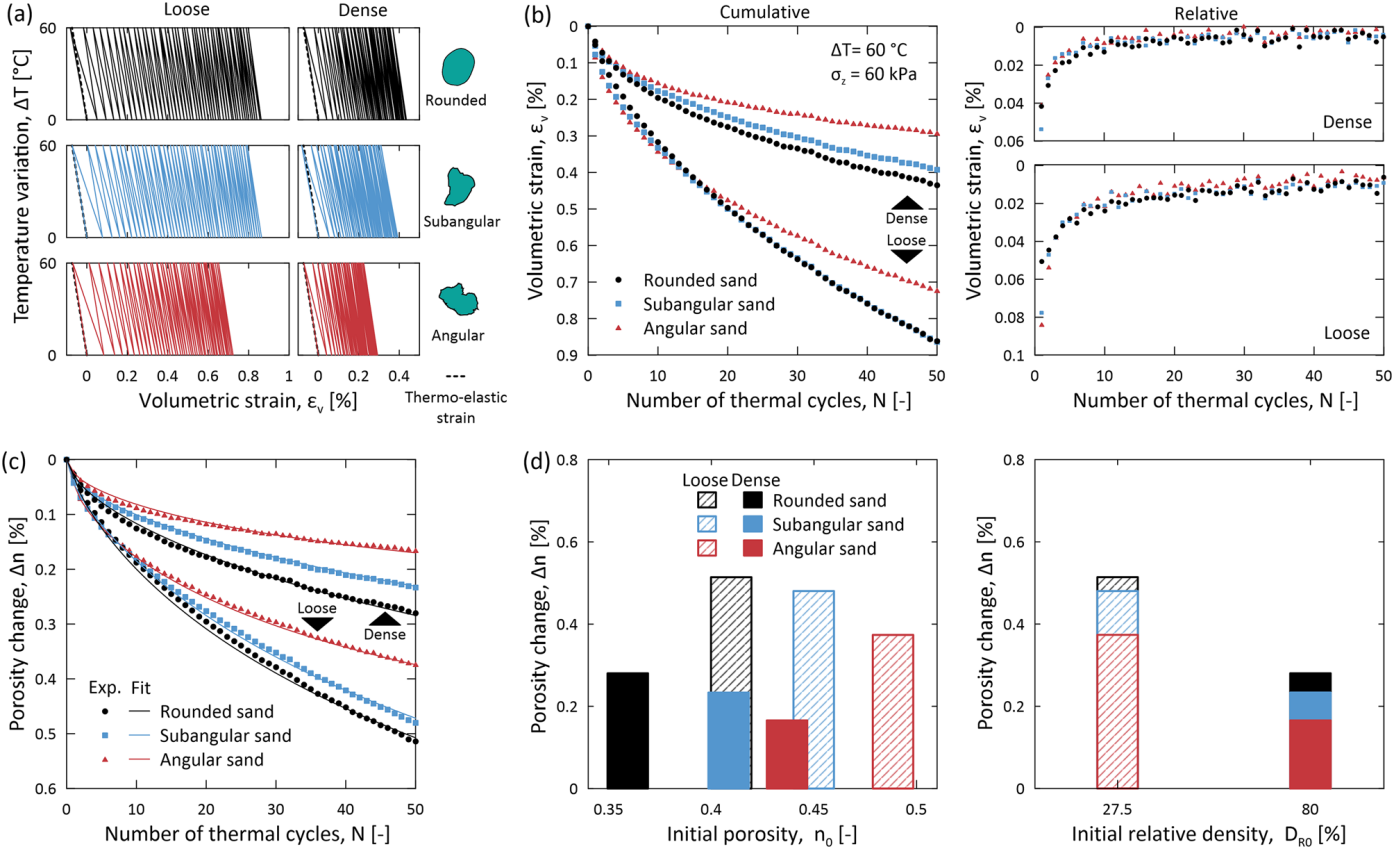
Relative density and particle shape effects in sands subjected to thermal cycling. Data refer to sands with rounded, subangular, and angular particle shapes subjected to 50 thermal cycles with an amplitude of $\Delta T=60$ °C at a constant vertical stress of ${\sigma }_{z}=60$ kPa. (**a**) Variation of volumetric strain ${\varepsilon }_{v}$ against temperature variation $\Delta T$ (representative particle shapes are drawn in green from images taken on actual particles). The dotted lines refer to the theoretical thermo-elastic volumetric strains characterizing the quartz that constitutes the particles of the tested granular materials and refer to a linear thermal expansion coefficient of $\alpha =1.22\times {10}^{-5}$ 1/°C (see “Methods”). (**b**) Cumulative and relative volumetric strains against number of thermal cycles $N$. (**c**) Evolution of porosity change $\Delta n$ with the number of applied thermal cycles. In addition to the experimental results, an empirical relationship to simulate the evolution of porosity changes over successive thermal cycles is presented (see ‘Methods”). (**d**) Changes in porosity $\Delta n$ as a function of initial porosity ${n}_{0}$ and initial relative density ${D}_{R0}$ after 50 thermal cycles.

Figure 1 shows the impacts of 50 thermal cycles on the volumetric strain and porosity of granular materials with variable relative densities and particle shapes. The results show that granular materials expand upon heating and contract upon cooling, undergoing a hysterical mechanical response due to thermal cycling (Fig. 1a). The slopes of the thermally induced strains upon heating are consistent with those predicted by the theory of thermo-elasticity. However, the slopes of the thermally induced strains upon cooling significantly deviate from the one predicted by the theory of thermo-elasticity, especially when considering the first cycles. Therefore, irreversible contractive deformations originate upon the completion of individual heating–cooling cycles, accumulating cycle after cycle (Fig. 1b). Notably, the slopes characterizing the thermally induced strains upon successive heating and cooling cycles tend to converge to the slope predicted by the theory of thermo-elasticity as the number of cycles increases, resulting in smaller irreversible deformations. This stabilization of volumetric strain also implies a concurrent stabilization of shear strain. This behavior aligns with the definition of plastic shakedown^38,39^ and, since it is caused by temperature variations, it is termed as thermally induced plastic shakedown, or simply thermal shakedown.

In agreement with previous evidence referring to sands subjected to one heating–cooling cycle^35,36^, the magnitude of contractive deformations affecting granular materials subjected to individual heating–cooling cycles is relatively limited. However, multiple thermal cycles result in significant bulk irreversible volumetric contractions of granular materials. Such deformations reach a cumulative value of about 0.9% after only 50 thermal cycles with an amplitude of 60 °C. Notably, deformations of about 1% are significant and can lead to problematic consequences for the operational performance of geotechnologies, such as geotechnical structures, geothermal systems, and underground thermal energy storage systems^13,40–42^. These deformations are also expected to contribute to changes in the morphology of landforms, not only in natural environments but also in built environments increasingly affected by temperature variations in the underground^43^.

The evolution of the porosity change of granular materials subjected to thermal cycling can be fitted with an empirical relationship (see Eq. (4) in “Methods”) previously proposed for describing the response of sands subjected to cyclic mechanical loads^38,39^ (Fig. 1c). The relatively consistent value of the exponent $m$ of such formulation across different tests suggests a universal underlying physics governing the phenomenon. In alignment with the available observations for sands undergoing cyclic mechanical loads, the considered empirical evolution law suggests the achievement of a “terminal density” in sands and other granular materials undergoing thermal shakedown (i.e., for a very large number of thermal cycles that cannot be achieved via laboratory experiments due to their daunting duration). Accordingly, it is postulated that the application of cyclic temperature variations to granular materials characterized by different initial porosities does not cause structural reorganizations that converge into a universal value of porosity when the number of thermal cycles tends to infinity, as this phenomenon does not characterize granular materials subjected to many cycles of mechanical loading and unloading ^38,39^.

The results unveil that deformations caused by thermal cycling markedly depend on the relative density of granular materials, even if the porosities of such materials differ dramatically at a given relative density. The porosity changes produced by 50 thermal cycles particularly indicate that irreversible deformations depend on the initial relative density, rather than the initial porosity (Fig. 1d). Dense sands exhibit smaller irreversible deformations than loose sands. This phenomenon results from the smaller potential for microstructural reorganizations that characterizes denser compared to looser granular materials.

The results further uncover that deformations caused by thermal cycling are strongly influenced by particle shape, with more significant deformations characterizing granular materials with increasingly rounded particles. Granular materials with rounded particles arguably undergo larger deformations compared to materials with angular particles due to the larger translational and rotational freedom of rounded particles compared to angular particles upon thermal cycling. Such a greater freedom is supported by the magnitude of plastic strains and porosity changes induced by thermal shakedown for each relative density. Exceptions apply to the first few thermal cycles, where granular materials with rounded particles show smaller plastic strains compared to the materials with subangular and angular particles, in agreement with previous results addressing the impacts of one heating–cooling cycle on the same materials^36^. In these circumstances, the larger initial porosity characteristic of sands with angular and subangular particles compared to sands with rounded particles is considered to overcome the inhibited freedom caused by particle angularity due to an initially higher potential for structural reorganizations.

**Figure 2:**
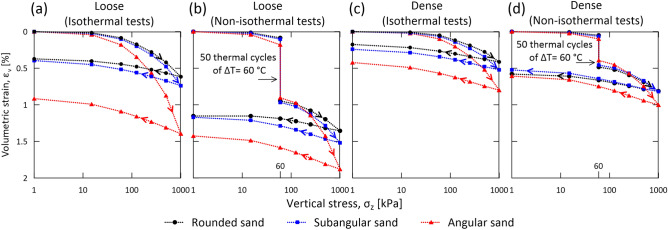
Relative density and particle shape effects in sands subjected to mechanical loading and thermal cycling. Volumetric strain ${\varepsilon }_{v}$ is plotted against the applied vertical stress ${\sigma }_{z}$. The response of sands subjected to 50 thermal cycles of $\Delta T=60$ °C under a constant applied stress of ${\sigma }_{z}=60$ kPa refers to the results shown in Fig. 1. The arrows indicated in the various curves shows the response of the material as the experiments progress over time. Response of loose sands in (**a**) isothermal tests and (**b**) non-isothermal tests. Response of dense sands in (**c**) isothermal tests and (**d**) non-isothermal tests.

Figure 2 shows the mechanical response of granular materials with irregular particle shapes before, during, and after thermal cycling by comparing the compression curves associated with the non-isothermal and isothermal tests. Both the isothermal and non-isothermal tests indicate that granular materials characterized by smaller relative densities exhibit larger deformations under the same applied mechanical load (Fig. 2a,c) or thermal load (Fig. 2b,d). Thermal cycling causes a progressive increase in contractive deformations under constant applied vertical stress (Fig. 2b,d). 50 thermal cycles of $\Delta T=60$ °C cause contractive strains whose magnitudes can vary from about half to a similar value of strains caused by the application of a vertical stress of ${\sigma }_{v}=1$ MPa. This evidence characterizes sands under both loose conditions (Fig. 2a,b) and dense conditions (Fig. 2c,d).

The obtained results allow to discover a peculiar role of particle shape on the deformation of granular materials, depending on whether mechanical or thermal loading is considered. Deformations caused by mechanical loading increase with the particle angularity (Fig. 2a,c). In other words, granular materials with angular particles deform more than materials with rounded particles under the same applied mechanical load, exhibiting a larger compressibility. In contrast, deformations caused by thermal loads decrease with the particle angularity (Fig. 2b,d). In other words, granular materials with angular particles deform less than materials with rounded particles upon thermal cycles of the same amplitude.

Therefore, particle shape involves complex effects on the mechanics of granular materials. An increasing particle angularity can lead to: (a) a higher porosity, (b) fewer but sharper contacts, and (c) particle interlocking due to concave surfaces. These aspects can lead to opposite consequences. Upon mechanical loading, effects (a) and (b) appear to govern the response of granular materials through a larger potential for plastic strains, as voids can be reduced when significant particle rearrangements occur. These effects may also justify the exceptions associated with the first few thermal cycles. Upon thermal loading, effects (b) and (c) appear to govern the response of granular materials, with a progressively hampered influence of effect (a) during successive thermal cycles. Rough and concave surfaces generate larger friction and rolling resistances between particles, which hamper their relative sliding and rolling. Therefore, after the first thermal cycles, increasingly restrained particle rearrangements characterize angular sands, resulting in smaller consequent deformations for multiple thermal cycles when compared to rounded sands.

### Stress level effects

This section further explores the impacts of thermal cycles on the mechanics of granular materials with reference to the influence of the stress level. This analysis resorts to the results of (1) isothermal tests and (2) non-isothermal tests involving the application of 50 thermal cycles with an identical amplitude of $\Delta T=60$ °C at variable stress levels of ${\sigma }_{v}=60$, $250$, and $500$ kPa. Results refer to the rounded sand. See “Methods” for details.

**Figure 3:**
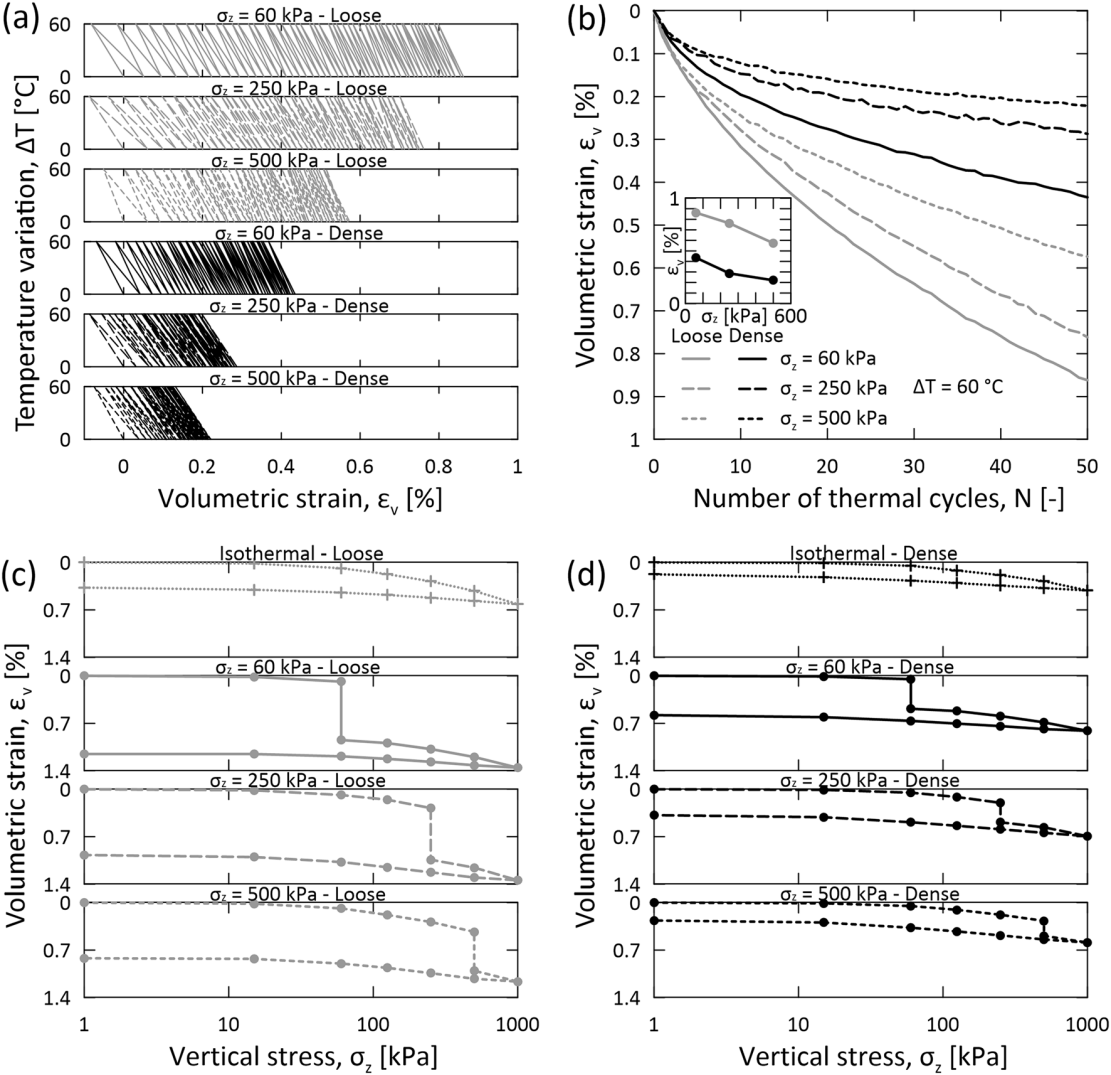
Stress level effects in sands subjected to mechanical loading and thermal cycling. Data refer to the rounded sand subjected to 50 thermal cycles with an amplitude of $\Delta T=60$ °C at a constant vertical stress of ${\sigma }_{z}=60$, 250, and $500$ kPa in the non-isothermal tests, as well as to mechanical loading at constant ambient temperature in the isothermal tests. (**a**) Variation of volumetric strain ${\varepsilon }_{v}$ against temperature variation $\Delta T$. (**b**) Cumulative volumetric strains ${\varepsilon }_{v}$ against the number of thermal cycles $N$. The embedded plot shows the correlation between the irreversible strain ${\varepsilon }_{v}$ (axis limit: 0–1%) after 50 cycles and vertical stress ${\sigma }_{z}$ (axis limit: 0–600 kPa). (**c**) Variation of volumetric strain ${\varepsilon }_{v}$ against vertical stress ${\sigma }_{z}$ in loose sands. (**d**) Variation of volumetric strain ${\varepsilon }_{v}$ against vertical stress ${\sigma }_{z}$ in dense sands.

Figure 3 shows the impacts of 50 thermal cycles applied at different stress levels on the volumetric strain of granular materials at two relative densities. The results consistently indicate a clear trend: a larger applied stress prior to thermal cycling leads to smaller thermally induced irreversible strains (Fig. 3a,b). Specifically, a non-linear, inverse relationship between the magnitude of the applied stress level and the irreversible strains caused by thermal cycling is observed. Consequently, a variable applied stress level involves a considerable difference in the magnitude of contractive volumetric strains caused by thermal cycling relative to those caused by mechanical loading (Fig. 3c,d).

The hampered magnitude of thermal shakedown at elevated levels of applied stress before thermal cycling is ascribed to the heightened structural stability observed in granular materials under higher stress conditions. This enhanced stability arguably arises from the strengthened force chains and larger restraint within the materials, which occur for increasing mechanical loads and material relative densities, respectively. The underlying rationale is that more heavily loaded and denser granular structures require more substantial perturbations to yield microstructural changes associated with thermal shakedown.

### Temperature amplitude effects

This section concludes the analysis of the impacts of thermal cycles on the mechanics of granular materials with reference to the influence of the temperature amplitude. This analysis resorts to the results of (1) isothermal tests and (2) non-isothermal tests involving the application of 50 thermal cycles with variable amplitudes of $\Delta T=30$, $45$, and $60$ °C at an identical stress level of ${\sigma }_{v}=60$ kPa. Results refer to the rounded sand. See “Methods” for details.

**Figure 4:**
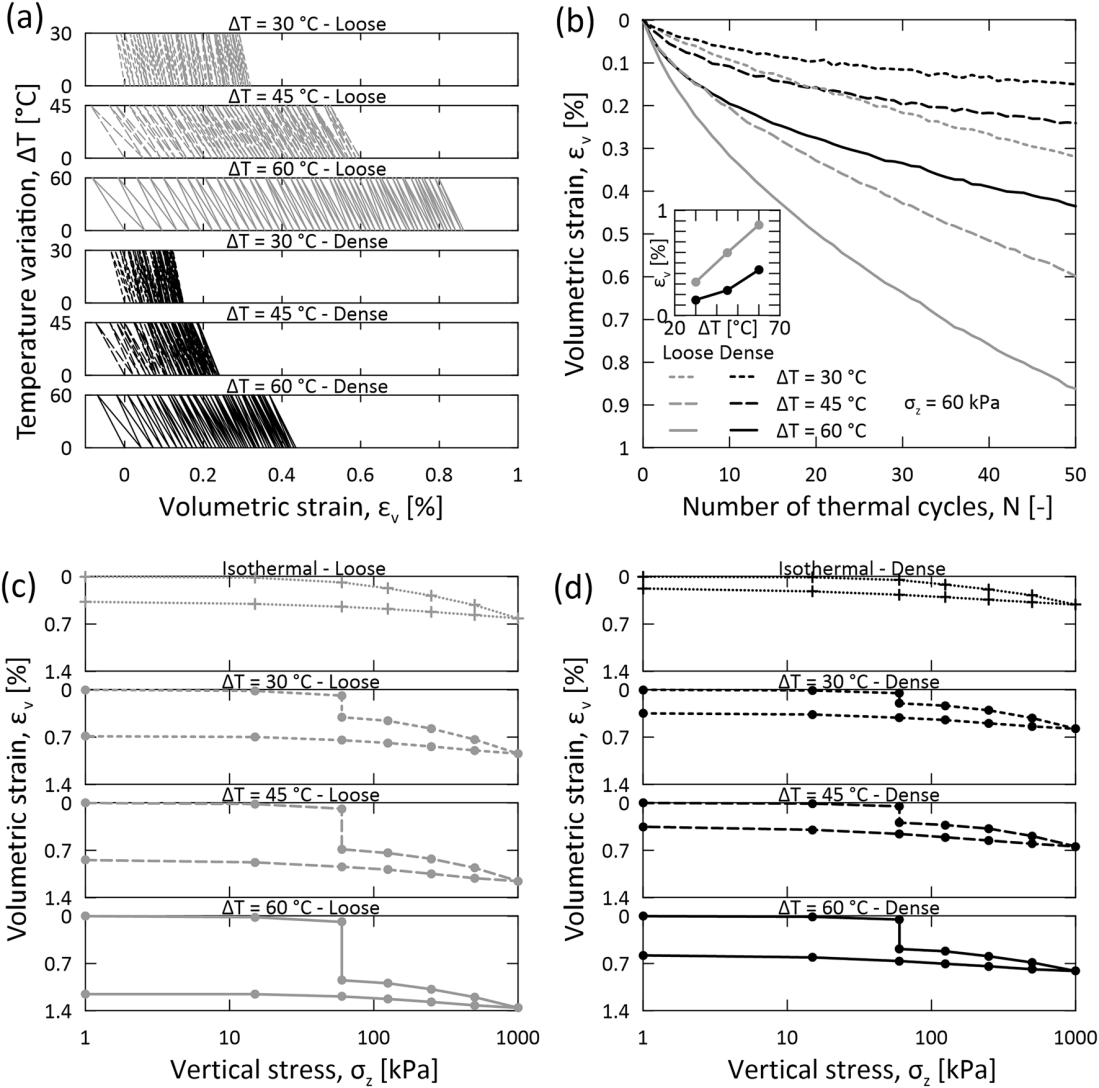
Temperature amplitude effects in sands subjected to mechanical loading and thermal cycling. Data refer to the rounded sand subjected to 50 thermal cycles with an amplitude of $\Delta T=30$, $45$, and $60$ °C at a constant vertical stress of ${\sigma }_{z}=60$ kPa in the non-isothermal tests, as well as to mechanical loading at constant ambient temperature in the isothermal tests. (**a**) Variation of volumetric strain ${\varepsilon }_{v}$ against temperature variation $\Delta T$. (**b**) Cumulative volumetric strains against number of thermal cycles $N$. The embedded plot shows the correlation between the irreversible strain ${\varepsilon }_{v}$ (axis limit: 0–1%) after 50 cycles and temperature amplitude $\Delta T$ (axis limit: 20–70 °C). (**c**) Variation of volumetric strain ${\varepsilon }_{v}$ against the vertical stress ${\sigma }_{z}$ in loose sands. (**d**) Variation of volumetric strain ${\varepsilon }_{v}$ against the vertical stress ${\sigma }_{z}$ in dense sands.

Figure 4 shows the impacts of 50 thermal cycles with different amplitudes on the volumetric strain of granular materials at two relative densities. The results consistently indicate another clear trend: thermal cycles with a larger temperature amplitude generate more significant thermally induced irreversible strains (Fig. 4a,b). Specifically, a non-linear relationship between temperature amplitude and irreversible strains is observed. As a result, doubling the amplitude of temperature variations from $30$ to $60$ °C results in more than proportional thermally induced strains. Under both loose and dense conditions, granular materials subjected to thermal cycles with an amplitude of $\Delta T=60$ °C show a more pronounced hysterical response (Fig. 4a) and larger plastic strains (Fig. 4b) compared to thermal cycles with an amplitude of $\Delta T=45$ and 30 °C. This result is consistent with previous evidence obtained for glass spheres^1^ and originates from more significant thermally induced particle deformations and interconnected variations in interparticle forces^44^. A variable temperature amplitude involves a significant change in the magnitude of contractive volumetric strains caused by thermal cycling relative to mechanical loading (Fig. 4c,d). This result highlights, once again, the considerable influence of thermal loading compared to mechanical loading.

## Discussion

This investigation explored various uncharted impacts of thermal cycling on the mechanics of granular materials, with a focus on sands. Experiments were performed to analyze the problem with reference to the following variables: (1) relative density, (2) particle shape, (3) stress level, and (4) temperature amplitude. With these premises, this study developed unprecedented knowledge on the role of variables (1–3) for the mechanics of granular materials under non-isothermal conditions, as this knowledge was previously available only for isothermal conditions. Additionally, this study provided further knowledge about variable (4). Relative densities and stress levels associated with depths ranging from a few meters to kilometers were addressed in light of the different behavior exhibited by looser or denser and more or less stressed granular materials under isothermal conditions, which also characterizes granular materials under non-isothermal conditions. Rounded, subangular, and angular particle shapes were considered in light of the highly variable shapes that are renowned to characterize sands and other granular materials in nature, and generate different mechanical responses of these materials under isothermal conditions, as also unveiled under non-isothermal conditions. Various temperature amplitudes were considered to explore the response of granular materials in multiple natural and engineered systems that undergo temperature variations.

The results of this work indicate that thermal cycles cause significant evolutionary impacts on the mechanics of granular materials. These evolutionary impacts are ruled by thermal shakedown: the accumulation of irreversible contractive bulk deformations of granular materials caused by cyclic temperature variations under a constant value of applied stress, which shall tend to stabilize as the number of thermal cycles approach infinity. Based on the results of this work, thermal shakedown characterizes granular materials irrespective of whether they are loose or dense, subjected to low or high stresses, characterized by rounded or angular particles, or undergoing thermal cycles of smaller or greater amplitudes. Such a phenomenon originates from thermally induced particle deformations, yielding irreversible strains after each heating–cooling cycle. These irreversible strains become smaller for successive thermal cycles but continue to accumulate over 50 cycles, resulting in bulk deformations comparable to those caused by substantial (monotonic) mechanical loads.

The results support that thermally induced deformations of sands caused by 50 thermal cycles can be significant enough to impair the serviceability of geotechnologies and change the morphology of landforms. Complementary results further suggest that sands previously subjected to a given number of thermal cycles undergo marked irreversible deformations when these materials are remolded and subjected again to a comparable number of thermal cycles (this evidence appears to hold at least as long as granular materials remain unaffected by substantial changes in their particle size, shape, or distribution, and are characterized by comparable initial conditions). In other words, perturbations that modify the structure of granular materials (e.g., rises in porosity) modify (e.g., increase) their potential to undergo particle rearrangements due to thermal cycling. Therefore, granular materials subjected to a myriad of thermal cycles, which would thus experience little irreversible strains due to individual heating–cooling cycles, may recursively undergo a surge in the magnitude of such strains if their structural reorganization potential is enhanced.

The consideration of more than 50 thermal cycles was not pursued in this work due to the lengthy experimental program resulting from the significant time (~ 15 days) required by each experiment to ensure rigorous control of the testing conditions and the choice to run multiple repeatability experiments for each combination of the studied parameters. However, the consideration of a larger number of thermal cycles would not have changed the fundamental understanding of the role of the studied variables. For example, the consideration of a medium dense sand, instead of the tested loose and dense sands, would have yielded strains of intermediate magnitude compared to those obtained in this work.

The findings of this study advance the understanding of thermal shakedown in sands by elucidating the influence of irregular particle shapes and other key physical variables on this phenomenon. The fundamental insights gleaned from this research have broader implications beyond sands and are applicable to a diverse array of granular materials with irregular particle shapes, including gravels, cobbles, regolith, polymers, and grains. Moreover, the results of this study not only provide inspiration but also serve as validation for future constitutive and computational models aimed at addressing the mechanics of granular materials under non-isothermal conditions. Ultimately, this contribution enhances scientific understanding and has practical implications for engineering and technology.

## Methods

### Experimental apparatus

The laboratory experiments at the basis of this work employ an advanced soil testing apparatus that allows to investigate the response of granular materials under laterally restrained (i.e., oedometric) conditions. Such conditions provide a simple yet representative and robust testing condition for the developed thermo–mechanical experiments. The features of this apparatus, manufactured by Wille Geotechnik (Germany), are detailed elsewhere^36^ and are only summarized hereafter for reference.

In the employed oedometer apparatus, cylindrical material samples can be subjected to any desired sequence of thermo-hydro-mechanical loading. In this work, the tested samples have an initial height of ${h}_{0}=18$ mm and diameter of $D=71.4$ mm. The apparatus allows controlling the applied vertical stress ${\sigma }_{v}$ via a force sensor and a loading frame. The lateral restraint for the sample is provided by a rigid ring made of invar. The temperature is controlled through a thermostat connected to the mechanical testing apparatus wherein silicone oil circulates around the sample, allowing to impose any desired profile of temperature variation $\Delta T$ to the sample. The sample vertical displacement $\Delta h$ is measured by two digital displacement sensors with high accuracy.

### Tested materials

The granular materials tested in this work consist of three types of dry silica sand: a rounded F-35 sand (Ottawa, IL), a subangular NJ #00 sand (Mauricetown, NJ), and an angular 1 Q-ROK sand (Berkeley Springs, WV). The properties of such materials are detailed elsewhere^36^ and are only summarized hereafter.

All the tested materials are composed by over 99% of quartz. They have a very similar particle size distribution but a very different particle shape, allowing to isolate the influence of the former variable for a detailed study of the latter variable. Figure S1 shows the particle size distribution of the tested materials obtained by a sieve analysis and qualitative features of the shape of their constituting particles obtained by scanning electron microscopy (SEM). The maximum void ratio ${e}_{max}$ and minimum void ratio ${e}_{min}$ of the considered materials (i.e., representing their loosest and densest states) were determined with reference to ASTM standards^45,46^. Table S1 summarizes these void ratios for the tested granular materials.

A characterization of the shape of the particles constituting the tested materials was provided by the material suppliers, but was also achieved as a part of the present experimental investigation following the method of Zhang and Hryciw ^47^. In this context, the roundness and sphericity of the particles were quantified. Roundness was defined as the ratio of the average radius of curvature of the corners of a particle to the radius of the maximum inscribed circle. Sphericity was defined as the ratio of particle projected area and minimum circumscribing circle. Around 150 particle projections for each type of material were captured by a microscope camera. Figure S2 shows the roundness and sphericity distributions of the three types of granular materials. The mean roundness is 0.610, 0.430, 0.356 for the rounded, subangular, angular sands, respectively, and their mean sphericity is 0.770, 0.660 and 0.659, respectively.

### Laboratory experiments

For this study, two classes of laboratory experiments were performed: (1) isothermal and (2) non-isothermal tests. Each isothermal test was performed three times to check repeatability. Each non-isothermal test was performed twice for the same purpose, primarily due to its long duration. The protocols underpinning these experiments are detailed hereafter.

Tests were performed on each type of granular material characterized by two values of relative density representative of loose conditions (${D}_{R} = 27.5\pm 2.5$%) and dense conditions (${D}_{R} = 80\pm 2.5$%). The loose samples were prepared by the air-pluviation method^48^. The dense samples were first air-pluviated and then densified by a vibratory table. The errors in relative density control were evaluated by analyzing the variations in more than 10 repeated sample preparations using the same methods.

The isothermal tests consisted in the mechanical loading and unloading of the tested materials at constant ambient temperature. The tests specifically involved a loading process from 1 to 1000 kPa and an unloading process from 1000 to 1 kPa, following incremental loading steps of 1, 15, 60, 125, 250, 500, and 1000 kPa for both loading and unloading (in reverse order). Each loading step was maintained for 30 min. The tests were performed at room temperature with no heating or cooling applied to the sample. The sample temperature always remained at around $22\pm 0.5$ °C throughout the tests. Figure S3a shows the schematic protocol considered for the isothermal tests. Figure S3b illustrates representative readings of vertical stress and sample temperature over time.

The non-isothermal tests consisted of a first phase of mechanical loading at constant ambient temperature, a second phase of thermal cycling at constant applied vertical stress, and subsequent phases of mechanical loading and unloading at constant ambient temperature. Specifically, each test involved a preloading process to reach a desired vertical stress level (60, 250, or 500 kPa), the application of 50 heating–cooling cycles of desired amplitude (30, 45, or 60 °C), post-cycling loading up to 1000 kPa, and unloading from 1000 to 1 kPa. Figure S4a,c,e show three examples of protocols employed in this study for thermal cycling tests considering temperature amplitudes of 30 and 60 °C, and stress levels 60 and 500 kPa. Figure S4b,d,f show representative readings of vertical stress and sample temperature over time. To accurately control the ambient sample temperature of 22 °C, the thermostat was employed to set the oil temperature at 21.7 °C. To impose temperature variations of 60, 45, and 30 °C, and thus achieve a sample temperature of 82, 67, and 52 °C, the thermostat was employed to set the oil temperature at 86.8, 70.5, and 54.2 °C, respectively. To ensure a steady and uniform temperature field within samples at each temperature step, the oil temperature was maintained at the setpoint temperature for 90 min. Figure S5 examines the readings obtained within one thermal cycle. Vertical stress fluctuates within ${\sigma }_{z}=60\pm 1$ kPa during the application of temperature variations characterized by an amplitude of $\Delta T=60$ °C, and the sample temperature is fully stabilized at the end of each temperature step. Thus, such protocol ensures optimal controls of both stress and temperature conditions.

### Useful definitions

In all tests, the volumetric strain is calculated as ${\varepsilon }_{v}=-\Delta h/{h}_{0}$ assuming zero lateral strains. Porosity is derived from the volumetric strain. Relative density is defined as:

1 $${D}_{R}=\frac{{e}_{ max}-e}{{e}_{max}-{e}_{ min}}\times 100\%$$

It expresses a measure of the current void ratio, $e$, in relation to the maximum void ratio, ${e}_{max}$, and the minimum void ratio, ${e}_{min}$.

### Reference thermo-elastic volumetric deformations

The magnitude of the observed thermally induced deformations during the application of the first temperature rise is compared with the magnitude of the thermo-elastic volumetric deformations of the material. These deformations are calculated by considering a temperature-dependent volumetric thermal expansion coefficient to provide more accurate results, rather than assuming a constant value for the volumetric thermal expansion coefficient. A detailed discussion on this subject is presented elsewhere^49^. The rate of volume change due to thermal expansion of sand particles can be expressed as $d{V}_{s}=\beta \left(T\right){V}_{s}dT$, where $\beta \left(T\right)$ is the theoretical volumetric thermal expansion coefficient. Exact integration provides the theoretical volume change with reference to the initial volume $\Delta V/{V}_{0}={\text{exp}}\left({\int }_{{T}_{i}}^{{T}_{f}}\beta \left(T\right)dT\right)-1$. Therefore, the equivalent volumetric thermal expansion coefficient of quartz is as follows:

2 $${\beta }^{eq}=\frac{{\text{exp}}\left({\int }_{{T}_{i}}^{{T}_{f}}\beta \left(T\right)dT\right)-1}{{T}_{f}-{T}_{i}}$$

Given the crystal structure of alpha quartz, the volumetric thermal expansion $\beta \left(T\right)$ is calculated as $\beta =2{\alpha }^{a}+{\alpha }^{c}$. Here, ${\alpha }^{a}$ represents the linear thermal expansion coefficient along the a-axis and ${\alpha }^{c}$ represents the one along the c-axis. Their values are determined by the 5th order polynomial expression provided by Kosinski et al.^50^ within the temperature range of − 50 to 150 °C. The expression of $\beta \left(T\right)$ is given by:

3 $$\beta \left(T\right)={a}_{0}+{a}_{1}T+{a}_{2}{T}^{2}+{a}_{3}{T}^{3}+{a}_{4}{T}^{4}+{a}_{5}{T}^{5}$$

with ${a}_{0}=3.33\times {10}^{-5}$ 1/°C, ${a}_{1}=6.94\times {10}^{-8}$ 1/°C^2^, ${a}_{2}=-1.667\times {10}^{-10}$ 1/°C^3^, ${a}_{3}=-6.094\times {10}^{-13}$ 1/°C^4^, ${a}_{4}=6.473\times {10}^{-15}$ 1/°C^5^, and ${a}_{5}=-1.304\times {10}^{-17}$ 1/°C^6^. During the heating process from an initial temperature ${T}_{i}=22$ °C to a final temperature ${T}_{f}=82$ °C, an equivalent ${\beta }^{eq}=3.65\times {10}^{-5}$ 1/°C can be obtained. A simplified average is considered in this study to account for the varying linear thermal expansion coefficients along different axes of the crystal structure of alpha quartz. Therefore, the deformations associated with the thermal expansion of the particles can be obtained with an equivalent linear thermal expansion coefficient of ${\alpha }^{eq}={\beta }^{eq}/3=1.22\times {10}^{-5}$ 1/°C.

### Porosity evolution law

The variation of the porosity of the tested materials upon the application of successive thermal cycles is compared with an empirical relationship between the porosity and the number of thermal cycles defined as:

4 $$\Delta n={n}_{T}+\left({n}_{1}-{n}_{T}\right){\left[1+{\left(\frac{i-1}{{N}^{*}}\right)}^{m}\right]}^{-1}-{n}_{0}$$

where ${n}_{T}$ is the terminal porosity as the number of cycles $i\to \infty$, ${n}_{1}$ is the porosity after $i=1$ cycle, ${N}^{*}$ is the characteristic number of cycles when considering ${1+N}^{*}$ as the required number of cycles for half of the total thermally induced compaction $({n}_{1}-{n}_{T})/2$ to occur, $m$ is a fitted exponent (with a relatively constant value $0.8\pm 0.05$ considered in this study), and ${n}_{0}$ is the initial porosity. Table S2 lists the parameters that are considered to fit the data in Fig. 1c.

### Experimental setup calibration

Extensive efforts have been made to calibrate the apparatus employed for the experiments reported in this work and correct its measurement from errors. Thermal losses were corrected in test protocols as shown in Fig. S4. The principal systematic error that needs to be corrected from the vertical displacement measurement on tested materials is the machine displacement ${\Delta h}_{machine}$, which can be induced by both mechanical and thermal perturbations. Therefore, calibration tests for each test protocol were performed to quantify the machine displacements at all stages. In these tests, the exact same loading paths applied to the tested materials as shown in Figs. S3 and S4 were applied to a dummy sample made of 316 stainless steel. The obtained machine displacements were corrected from the measurements of real material tests as discussed elsewhere^36^.

### Experimental reproducibility

Striving to achieve the greatest reliability of experimental measurements, the developed tests were performed using methodologies that previously showed high accuracy (i.e., trueness and precision) and the capability to quantify with statistical significance the impacts of thermal and mechanical loads on the mechanics of granular materials^36^. All attempts to replicate the tendencies in this study were successful.

### Supplementary Information

Supplementary Information.

## Data Availability

Correspondence and requests for materials should be addressed to AFRL. Processed data available on request from AFRL.
