# Author Correction: Thermal shakedown in granular materials with irregular particle shapes

**DOI:** 10.1038/s41598-024-60963-1

**Published:** 2024-05-06

**Authors:** Yize Pan, Xiaohui Gong, Alessandro F. Rotta Loria

**Affiliations:** Department of Civil and Environmental Engineering, Subsurface Opportunities and Innovations Laboratory, Northwestern University, Evanston, USA

Correction to: *Scientific Reports* 10.1038/s41598-024-57503-2, published online 21 March 2024

The original version of this Article contained an error in the Supplementary Information File, where Table S2 contained incorrect values in the parameters ‘${n}_{T}$ [-]’, ${^{\prime}N}^{*}$ [-]’ and ‘$m$ [-]’.

The incorrect Supplementary Information file is provided below.

The original Article has been corrected.

## Supplementary Information

Supplementary Information.

