# Supplementary material for: Thermal shakedown in granular materials with irregular particle shapes
Source: Sci Rep. 2024 Mar 21;14:6828. doi: 10.1038/s41598-024-57503-2 (PMC10957884; doi:10.1038/s41598-024-57503-2)
Supplement: Supplementary file 1 — Supplementary Information. [file 41598_2024_57503_MOESM1_ESM.docx]

**Thermal shakedown in granular materials with irregular particle shapes**

Yize Pan^1^, Xiaohui Gong^1^, Alessandro F. Rotta Loria^1*^

^1^ Northwestern University, Department of Civil and Environmental Engineering, Subsurface Opportunities and Innovations Laboratory

*^*^*Corresponding author: af-rottaloria@northwestern.edu

**Supplementary Material**

Table S1. Maximum and minimum void ratios of the three types of tested materials.

| Tested materials | Maximum void ratio $e_{max}$ [-] | Minimum void ratio $e_{min}$ [-] |
| --- | --- | --- |
| F-35 sand | 0.764 | 0.510 |
| NJ #00 sand | 0.995 | 0.642 |
| 1 Q-ROK sand | 1.049 | 0.708 |

Table S2. Estimated terminal porosity $\boldsymbol{n}_{\boldsymbol{T}}$ and fitted parameters of the empirical relationship.

| Particle shape and relative density | $n_{T}$ [-] | $N^{*}$ [-] | $m$ [-] |
| --- | --- | --- | --- |
| Rounded, loose | 0.396 | 95 | 0.8 |
| Rounded, dense | 0.3493 | 180 | 0.75 |
| Subangular, Loose | 0.4374 | 90 | 0.85 |
| Subangular, Dense | 0.4 | 200 | 0.75 |
| Angular, Loose | 0.4795 | 70 | 0.75 |
| Angular, Dense | 0.4325 | 80 | 0.75 |


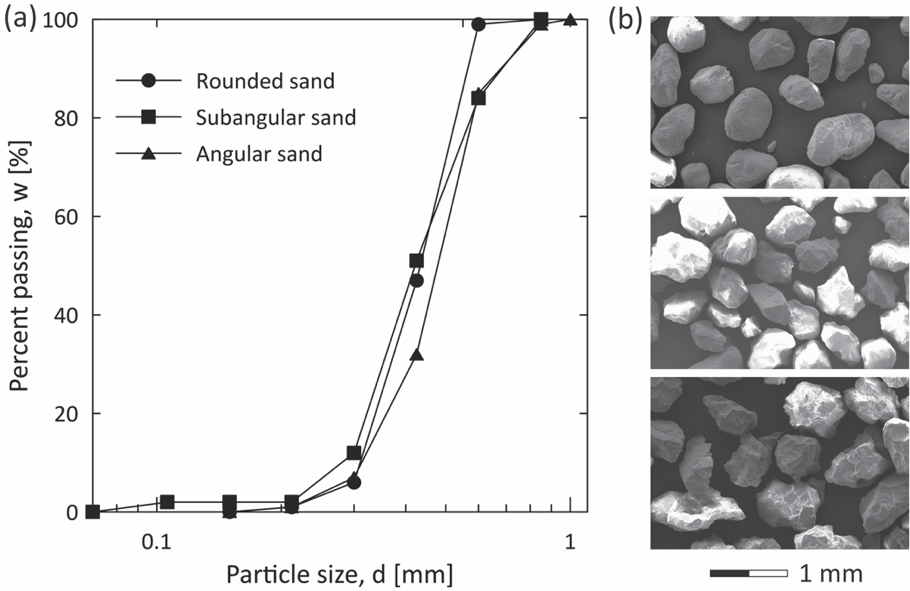


Fig. S1. Particle characteristics of the tested granular materials. (a) Particle size distribution and (b) SEM images of the rounded, subangular, angular sands (from top to bottom).


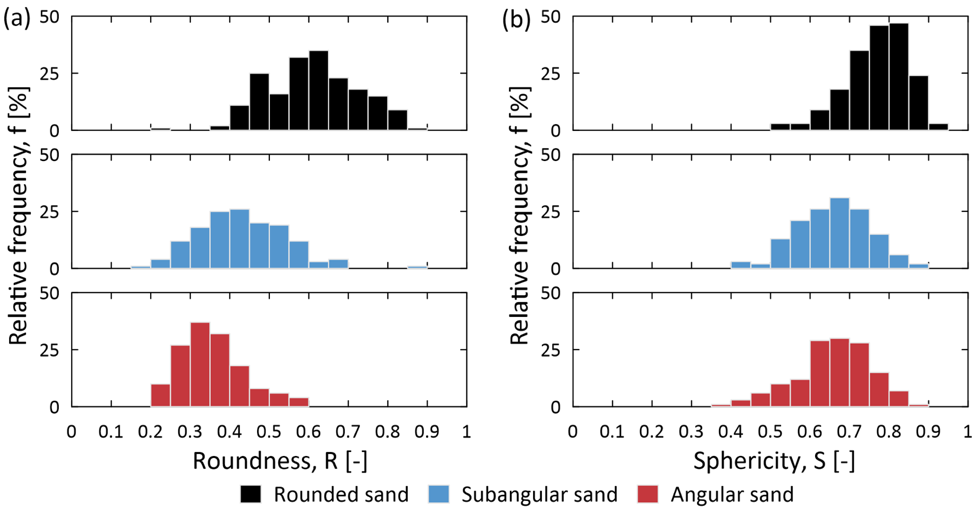


Fig. S2. Particle shape characterizations of the tested granular materials. (a) Histogram of roundness, $\boldsymbol{R}$. (b) Histogram of sphericity, $\boldsymbol{S}$.


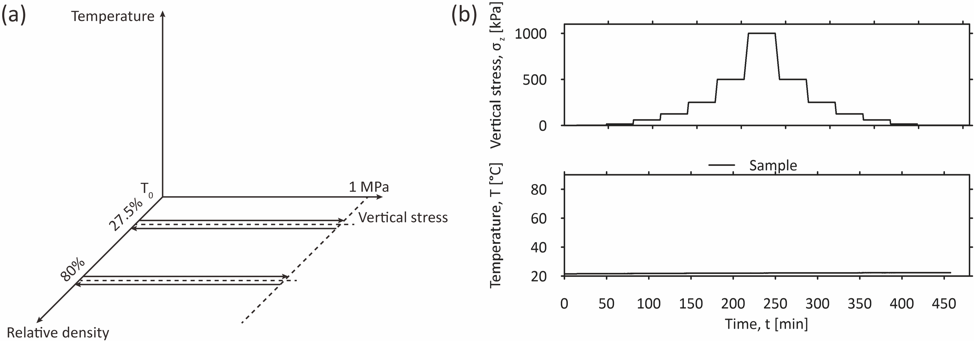


Fig. S3. Test protocol for the isothermal tests. (a) Loading paths (the variation of sample relative density $\boldsymbol{D}_{\boldsymbol{R}}$ is not represented for simplicity and clarity). (b) Prototype readings of vertical stress $\boldsymbol{\sigma}_{\boldsymbol{z}}$ and sample temperature $\boldsymbol{T}$ over time $\boldsymbol{t}$.


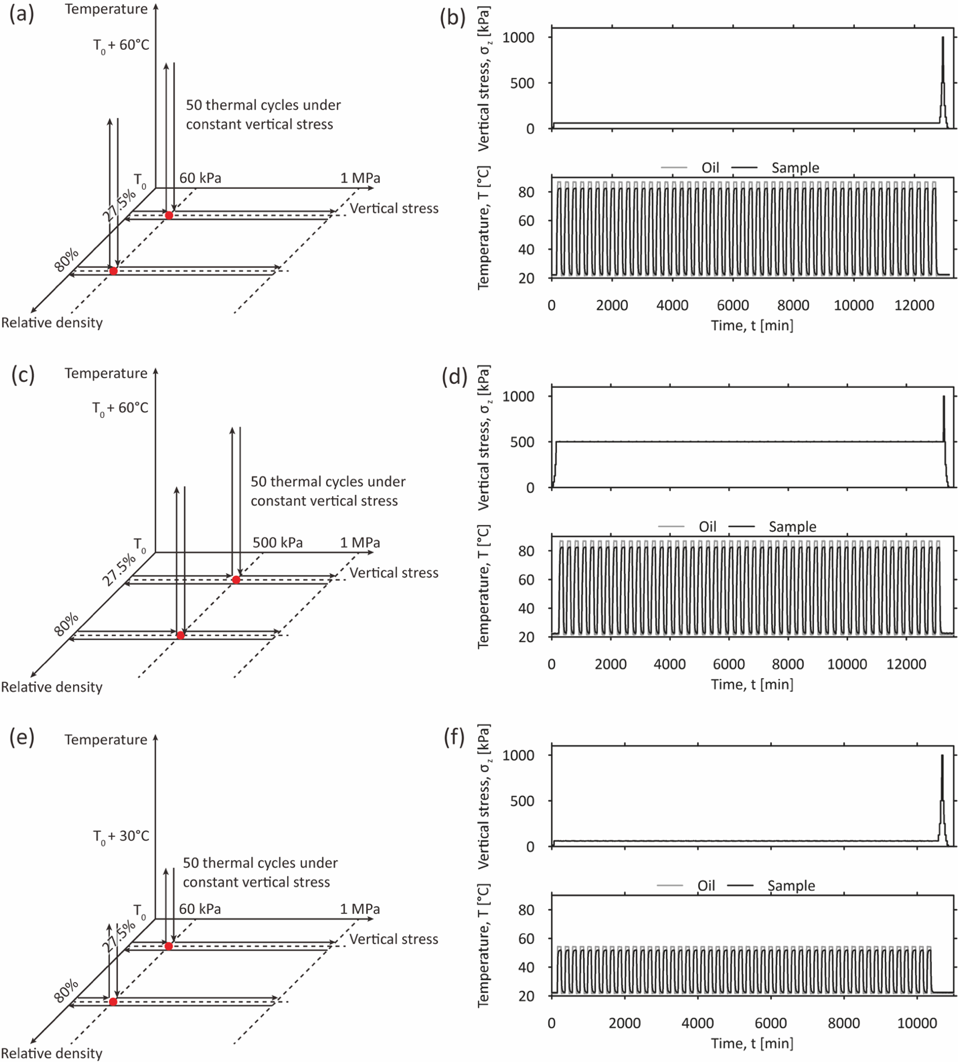


Fig. S4. Test protocols for selected non-isothermal tests considering different temperature amplitudes $\boldsymbol{\Delta T}$ and stress levels $\boldsymbol{\sigma}_{\boldsymbol{z}}$. Mechanical loading and unloading follow the same stress steps and time interval shown in Fig. S3. The variation of sample relative density $\boldsymbol{D}_{\boldsymbol{R}}$ is neglected for simplicity and clarity. Red dots mark the stress levels at which thermal cycling is applied. (a) Theoretical paths and (b) actual readings of vertical stress $\boldsymbol{\sigma}_{\boldsymbol{z}}$ and temperature $\boldsymbol{T}$ over time $\boldsymbol{t}$ considering $\boldsymbol{\Delta T=60}$°C and $\boldsymbol{\sigma}_{\boldsymbol{z}}\boldsymbol{=60}$ kPa. (c) Theoretical paths and (d) actual readings of vertical stress $\boldsymbol{\sigma}_{\boldsymbol{z}}$ and temperature $\boldsymbol{T}$ over time $\boldsymbol{t}$ considering $\boldsymbol{\Delta T=60}$°C and $\boldsymbol{\sigma}_{\boldsymbol{z}}\boldsymbol{=500}$ kPa. (e) Theoretical paths and (f) actual readings of vertical stress $\boldsymbol{\sigma}_{\boldsymbol{z}}$ and temperature $\boldsymbol{T}$ over time $\boldsymbol{t}$ considering $\boldsymbol{\Delta T=30}$°C and $\boldsymbol{\sigma}_{\boldsymbol{z}}\boldsymbol{=60}$ kPa.


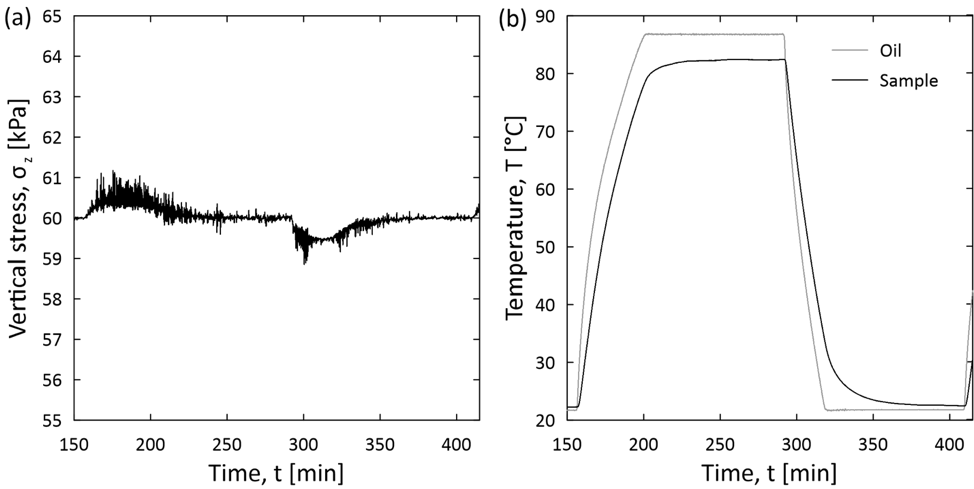


Fig. S5. Detailed view of vertical stress $\boldsymbol{\sigma}_{\boldsymbol{z}}$ and temperature $\boldsymbol{T}$ readings during one thermal cycle with a temperature amplitude of $\boldsymbol{\Delta T=60}$°C. The example represents the readings between $\boldsymbol{t =}$ 150 and 415 min in Figure S4(b). (a) Readings of vertical stress $\boldsymbol{\sigma}_{\boldsymbol{z}}$ during one thermal cycle. (b) Readings of temperature $\boldsymbol{T}$ during one thermal cycle.
